# *N*-[2-(2-Bromo­benzyl­amino)phen­yl]-*N*-butylformamide

**DOI:** 10.1107/S1600536809042883

**Published:** 2009-10-23

**Authors:** Sudesh T. Manjare, Ray J. Butcher, Harkesh B. Singh

**Affiliations:** a Department of Chemistry, Indian Institute of Technology Bombay, Powai, Mumbai 400 076, India; b Department of Chemistry, Howard University, 525 College Street NW, Washington DC 20059, USA

## Abstract

The title compound, C_18_H_21_BrN_2_O, crystallizes with two mol­ecules (*A* and *B*) in the asymmetric unit (*Z*′ = 2). The major differences between the two mol­ecules are related to the conformations adopted by their *n*-butyl side chains. The phenyl rings in both mol­ecules are almost perpendicular, making dihedral angles of 79.2 (3) and 80.8 (3)°. The amide units are planar (r.m.s. deviations of 0.0018 and 0.021 Å) and almost perpendicular to the phenyl rings to which they are attached [dihedral angles of 68.9 (4) and 71.1 (4)°]. In the crystal, molecules *A* and *B* each form only an intermolecular N—H⋯O hydrogen bond with an adjacent molecule of the same kind. There are no significant intermolecular interactions between molecules *A* and *B*.

## Related literature

For a related structure, see: Manjare *et al.* (2009 ▶). For related synthetic studies, see: Albéniz *et al.* (2002 ▶); Denk *et al.* (2001 ▶); Jarrar & Fataftah (1977 ▶); Çetinkaya *et al.* (1998 ▶).
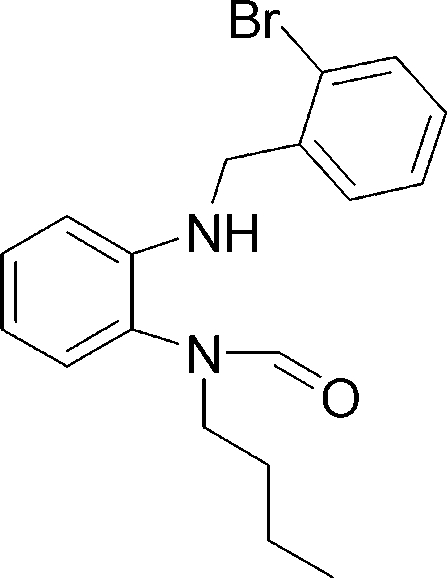


## Experimental

### 

#### Crystal data

C_18_H_21_BrN_2_O
*M* *_r_* = 361.28
Triclinic, 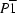

*a* = 8.7067 (4) Å
*b* = 10.8208 (6) Å
*c* = 19.7480 (13) Å
α = 87.180 (5)°
β = 82.622 (5)°
γ = 66.706 (5)°
*V* = 1694.72 (17) Å^3^
*Z* = 4
Mo *K*α radiation
μ = 2.43 mm^−1^
*T* = 200 K
0.49 × 0.28 × 0.23 mm

#### Data collection

Oxford Diffraction Gemini R diffractometer
Absorption correction: multi-scan (*CrysAlis RED*; Oxford Diffraction, 2009 ▶) *T*_min_ = 0.478, *T*_max_ = 1.000
13877 measured reflections
5942 independent reflections
3799 reflections with *I* > 2σ(*I*)
*R*_int_ = 0.049

#### Refinement

*R*[*F* ^2^ > 2σ(*F* ^2^)] = 0.095
*wR*(*F* ^2^) = 0.252
*S* = 1.10
5942 reflections
399 parameters
H-atom parameters constrained
Δρ_max_ = 1.94 e Å^−3^
Δρ_min_ = −0.82 e Å^−3^

### 

Data collection: *CrysAlis CCD* (Oxford Diffraction, 2009 ▶); cell refinement: *CrysAlis RED* (Oxford Diffraction, 2009 ▶); data reduction: *CrysAlis RED*; program(s) used to solve structure: *SHELXS97* (Sheldrick, 2008 ▶); program(s) used to refine structure: *SHELXL97* (Sheldrick, 2008 ▶); molecular graphics: *SHELXTL* (Sheldrick, 2008 ▶); software used to prepare material for publication: *SHELXTL*.

## Supplementary Material

Crystal structure: contains datablocks I, New_Global_Publ_Block. DOI: 10.1107/S1600536809042883/ds2009sup1.cif

Structure factors: contains datablocks I. DOI: 10.1107/S1600536809042883/ds2009Isup2.hkl

Additional supplementary materials:  crystallographic information; 3D view; checkCIF report

## Figures and Tables

**Table 1:** Hydrogen-bond geometry (Å, °)

*D*—H⋯*A*	*D*—H	H⋯*A*	*D*⋯*A*	*D*—H⋯*A*
N2*A*—H2*AA*⋯O1*A*^i^	0.88	2.15	2.947 (9)	150
N2*B*—H2*BA*⋯O1*B*^ii^	0.88	2.18	3.020 (10)	159

Symmetry codes: (i) 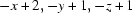; (ii) 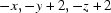.

**Fig. 1.**
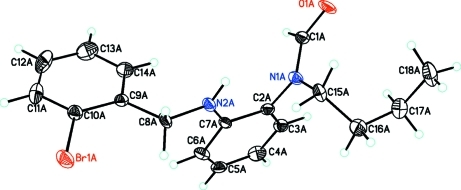
The molecular structure of C18H21BrN2O the showing the atom numbering scheme and 50% probability displacement ellipsoids.

**Fig. 2.**
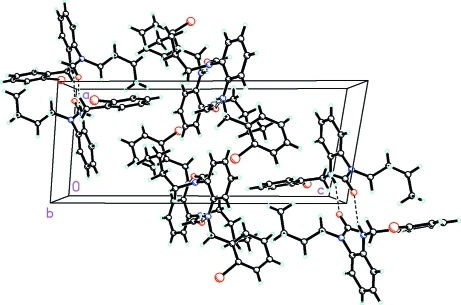
The molecular packing for C18H21BrN2O viewed down the b axis. The hydrogen bonding between N—H···N is shown by dashed lines.

**Fig. 3.**
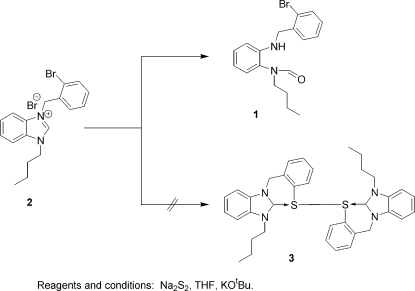
Preparation of the title compound.

## supplementary crystallographic information

### Comment

The structure of the title compound, (I), is shown below. Dimensions are available in the archived CIF.

The title compound, C_18_H_21_BrN_2_O, was obtained as a hydrolysis product during an attempted synthesis of the butyl substituted thiocarbene compound from the corresponding benzimidazolium salt (see Fig. 3). The compound crystallizes with two molecules in the asymmetric unit (*Z*' = 2). The major differences between the two molecules are related to the conformations adopted by the *n*-butyl substituents. This is shown most clearly by a comparison of the *n*-butyl torsion angles. In molecule A the torsion angles for N1A—C15A—C16A—C17A and C15A—C16A—C17A—C18A are 69.2 (11) and 66.8 (13)° while for molecule B the corresponding angles are 176.50 (10) and -61.8 (17)°.

The two phenyl rings in A and B make dihedral angles of 79.2 (3) and 80.8 (3), respectively with each other. Both molecules A and B form only an intermolecular N–H···O hydrogen bond with an adjoining molecule of the same kind. There are no significant intermolecular interactions between molecule A and B. The atoms making up the amide group for both A and B are planar (r.m.s. deviation of fitted atoms 0.0018 and 0.021 Å respectively) and both groups are almost perpendicular to the plane of the phenyl ring to which they are attached (dihedral angles of 68.9 (4) and 71.1 (4)° respectively).

While there has only been one structure reported for a related molecule (Manjare *et al.* 2009), there have been several synthetic studies reported (Jarrar & Fataftah, 1977; Denk *et al.* 2001; Çetinkaya *et al.* 1998; Albéniz *et al.* 2002).

### Experimental

The benzylimidazoylium salt 2 (1.0 mmol) was added to the solution of Na_2_S_2_ (2.0 mmol) at room temperature under nitrogen atmosphere and the reaction mixture was stirred for 6–10 h at room temperature as shown in scheme 1. Then KO*^t^*Bu (1.0 mmol) was added to the reaction mixture and stirred further for 5–7 h. After completion of reaction, the reaction was quenched by adding water (50 ml), and extracted with dichloromethane, dried over Na_2_SO_4_, and evaporated. The residue obtained was dissolved in toluene and small amount of petroleum ether (60–80 °C) was added to separate the residue from the solution. The solution was filtered and evaporated; the residue was dissolved in diethyl ether and a small amount of petroleum ether (60–80 °C) was added and the solution was kept on table to afford X-ray quality crystals of 1 as a minor product.

### Refinement

The crystal was weakly diffracting and no meaningful data could be obtained above a 2-theta of 50° so a high resolution cutoff was carried out with a threshold of d = 0.835. H atoms were placed in geometrically idealized positions and constrained to ride on their parent atoms with C—H distances of 0.95 and 0.99 Å *U*_iso_(H) = 1.2*U*_eq_(C) = [1.5*U*_eq_(CH_3_)]. The H attached to N was idealized with a distance of 0.88 Å. In the final difference Fourier there was a peak of 1.94 e/Å^3^ close to the Br atom.

### Crystal data

**Table:**

C_18_H_21_BrN_2_O	*Z* = 4
*M**_r_* = 361.28	*F*(000) = 744
Triclinic, *P*1	*D*_x_ = 1.416 Mg m^−^^3^
Hall symbol: -P 1	Mo *K*α radiation, λ = 0.71073 Å
*a* = 8.7067 (4) Å	Cell parameters from 5504 reflections
*b* = 10.8208 (6) Å	θ = 4.6–34.7°
*c* = 19.7480 (13) Å	µ = 2.43 mm^−^^1^
α = 87.180 (5)°	*T* = 200 K
β = 82.622 (5)°	Thick needle, colorless
γ = 66.706 (5)°	0.49 × 0.28 × 0.23 mm
*V* = 1694.72 (17) Å^3^	

### Data collection

**Table:**

Oxford Diffraction Gemini R diffractometer	5942 independent reflections
Radiation source: Enhance (Mo) X-ray Source	3799 reflections with *I* > 2σ(*I*)
graphite	*R*_int_ = 0.049
Detector resolution: 10.5081 pixels mm^-1^	θ_max_ = 25.2°, θ_min_ = 4.6°
ω scans	*h* = −10→10
Absorption correction: multi-scan (*CrysAlis RED*; Oxford Diffraction, 2009)	*k* = −12→12
*T*_min_ = 0.478, *T*_max_ = 1.000	*l* = −20→23
13877 measured reflections	

### Refinement

**Table:**

Refinement on *F*^2^	Primary atom site location: structure-invariant direct methods
Least-squares matrix: full	Secondary atom site location: difference Fourier map
*R*[*F*^2^ > 2σ(*F*^2^)] = 0.095	Hydrogen site location: inferred from neighbouring sites
*wR*(*F*^2^) = 0.252	H-atom parameters constrained
*S* = 1.10	*w* = 1/[σ^2^(*F*_o_^2^) + (0.088*P*)^2^ + 17.292*P*] where *P* = (*F*_o_^2^ + 2*F*_c_^2^)/3
5942 reflections	(Δ/σ)_max_ < 0.001
399 parameters	Δρ_max_ = 1.94 e Å^−^^3^
0 restraints	Δρ_min_ = −0.81 e Å^−^^3^

### Special details

**Table:**

Geometry. All e.s.d.'s (except the e.s.d. in the dihedral angle between two l.s. planes) are estimated using the full covariance matrix. The cell e.s.d.'s are taken into account individually in the estimation of e.s.d.'s in distances, angles and torsion angles; correlations between e.s.d.'s in cell parameters are only used when they are defined by crystal symmetry. An approximate (isotropic) treatment of cell e.s.d.'s is used for estimating e.s.d.'s involving l.s. planes.
Refinement. Refinement of *F*^2^ against ALL reflections. The weighted *R*-factor *wR* and goodness of fit *S* are based on *F*^2^, conventional *R*-factors *R* are based on *F*, with *F* set to zero for negative *F*^2^. The threshold expression of *F*^2^ > σ(*F*^2^) is used only for calculating *R*-factors(gt) *etc*. and is not relevant to the choice of reflections for refinement. *R*-factors based on *F*^2^ are statistically about twice as large as those based on *F*, and *R*- factors based on ALL data will be even larger.

### Fractional atomic coordinates and isotropic or equivalent isotropic displacement parameters (Å^2^)

**Table:**

	*x*	*y*	*z*	*U*_iso_*/*U*_eq_	
Br1A	0.37929 (12)	1.19458 (9)	0.60470 (6)	0.0425 (3)	
O1A	1.2053 (8)	0.4014 (6)	0.4756 (4)	0.0476 (19)	
N1A	1.1593 (9)	0.6243 (7)	0.4840 (4)	0.0288 (16)	
N2A	0.8719 (8)	0.8358 (7)	0.5427 (4)	0.0299 (17)	
H2AA	0.8683	0.7671	0.5218	0.036*	
C1A	1.2027 (11)	0.4980 (8)	0.5061 (5)	0.033 (2)	
H1AA	1.2355	0.4810	0.5509	0.039*	
C2A	1.1740 (10)	0.7226 (8)	0.5264 (5)	0.0264 (19)	
C3A	1.3288 (11)	0.7153 (9)	0.5390 (5)	0.031 (2)	
H3AA	1.4269	0.6425	0.5202	0.037*	
C4A	1.3465 (12)	0.8090 (10)	0.5775 (5)	0.041 (2)	
H4AA	1.4549	0.8013	0.5860	0.050*	
C5A	1.2051 (13)	0.9140 (9)	0.6036 (5)	0.037 (2)	
H5AA	1.2165	0.9800	0.6302	0.044*	
C6A	1.0472 (12)	0.9271 (9)	0.5925 (5)	0.033 (2)	
H6AA	0.9518	1.0023	0.6110	0.040*	
C7A	1.0242 (10)	0.8308 (8)	0.5541 (4)	0.0233 (18)	
C8A	0.7142 (11)	0.9480 (9)	0.5629 (5)	0.032 (2)	
H8AA	0.6292	0.9468	0.5344	0.039*	
H8AB	0.7308	1.0327	0.5530	0.039*	
C9A	0.6447 (10)	0.9492 (8)	0.6369 (5)	0.0285 (19)	
C10A	0.4963 (11)	1.0512 (9)	0.6642 (5)	0.032 (2)	
C11A	0.4255 (13)	1.0523 (12)	0.7299 (5)	0.045 (3)	
H11A	0.3235	1.1241	0.7464	0.054*	
C12A	0.5068 (16)	0.9449 (14)	0.7730 (5)	0.059 (3)	
H12A	0.4588	0.9425	0.8188	0.070*	
C13A	0.6551 (16)	0.8438 (13)	0.7486 (6)	0.056 (3)	
H13A	0.7114	0.7715	0.7775	0.067*	
C14A	0.7229 (13)	0.8476 (10)	0.6812 (5)	0.038 (2)	
H14A	0.8270	0.7776	0.6651	0.046*	
C15A	1.1081 (11)	0.6630 (9)	0.4164 (5)	0.032 (2)	
H15A	1.0037	0.7458	0.4202	0.039*	
H15B	1.0819	0.5910	0.3980	0.039*	
C16A	1.2394 (13)	0.6877 (10)	0.3668 (6)	0.045 (2)	
H16A	1.2736	0.7527	0.3878	0.054*	
H16B	1.1871	0.7307	0.3255	0.054*	
C17A	1.3970 (14)	0.5643 (12)	0.3448 (6)	0.050 (3)	
H17A	1.4804	0.5943	0.3182	0.060*	
H17B	1.4462	0.5180	0.3861	0.060*	
C18A	1.3692 (18)	0.4668 (14)	0.3032 (7)	0.074 (4)	
H18A	1.4779	0.3957	0.2870	0.111*	
H18B	1.3122	0.5130	0.2640	0.111*	
H18C	1.2991	0.4269	0.3312	0.111*	
Br1B	0.13415 (14)	0.30839 (10)	0.89286 (6)	0.0507 (4)	
O1B	0.1010 (8)	1.1026 (6)	1.0104 (4)	0.0420 (17)	
N2B	0.2350 (10)	0.6702 (8)	0.9504 (5)	0.043 (2)	
H2BA	0.1516	0.7404	0.9700	0.051*	
N1B	0.2818 (10)	0.8840 (8)	1.0051 (4)	0.0367 (19)	
C1B	0.2085 (12)	1.0049 (9)	0.9810 (6)	0.040 (2)	
H1B	0.2438	1.0180	0.9347	0.048*	
C2B	0.4187 (11)	0.7787 (9)	0.9663 (4)	0.029 (2)	
C3B	0.5739 (12)	0.7863 (10)	0.9563 (5)	0.040 (2)	
H3BA	0.5885	0.8603	0.9741	0.048*	
C4B	0.7098 (13)	0.6858 (11)	0.9199 (6)	0.046 (3)	
H4BA	0.8176	0.6899	0.9127	0.056*	
C5B	0.6836 (12)	0.5805 (11)	0.8948 (5)	0.045 (3)	
H5BA	0.7750	0.5102	0.8704	0.054*	
C6B	0.5278 (12)	0.5753 (10)	0.9044 (5)	0.040 (2)	
H6BA	0.5135	0.5016	0.8859	0.048*	
C7B	0.3897 (11)	0.6742 (9)	0.9403 (5)	0.034 (2)	
C8B	0.1972 (13)	0.5605 (10)	0.9317 (5)	0.042 (2)	
H8BA	0.0930	0.5649	0.9602	0.051*	
H8BB	0.2892	0.4758	0.9432	0.051*	
C9B	0.1733 (11)	0.5527 (10)	0.8569 (5)	0.039 (2)	
C10B	0.1435 (11)	0.4489 (9)	0.8321 (5)	0.036 (2)	
C11B	0.1136 (14)	0.4417 (12)	0.7659 (6)	0.052 (3)	
H11B	0.0909	0.3685	0.7518	0.063*	
C12B	0.1169 (16)	0.5430 (14)	0.7193 (7)	0.063 (3)	
H12B	0.0989	0.5396	0.6731	0.076*	
C13B	0.1474 (15)	0.6475 (14)	0.7431 (6)	0.061 (4)	
H13B	0.1470	0.7185	0.7128	0.074*	
C14B	0.1787 (14)	0.6529 (11)	0.8103 (7)	0.055 (3)	
H14B	0.2039	0.7248	0.8243	0.066*	
C15B	0.2170 (14)	0.8513 (11)	1.0754 (5)	0.046 (3)	
H15C	0.0960	0.9090	1.0858	0.055*	
H15D	0.2295	0.7562	1.0768	0.055*	
C16B	0.3143 (18)	0.8742 (17)	1.1276 (7)	0.074 (4)	
H16C	0.3079	0.9677	1.1241	0.089*	
H16D	0.4342	0.8122	1.1189	0.089*	
C17B	0.240 (2)	0.8494 (17)	1.2001 (7)	0.077 (4)	
H17C	0.2473	0.7556	1.2028	0.093*	
H17D	0.3088	0.8596	1.2338	0.093*	
C18B	0.064 (2)	0.9411 (16)	1.2183 (7)	0.089 (5)	
H18D	0.0199	0.9132	1.2619	0.133*	
H18E	−0.0034	0.9385	1.1827	0.133*	
H18F	0.0576	1.0329	1.2226	0.133*	

### Atomic displacement parameters (Å^2^)

**Table:**

	*U*^11^	*U*^22^	*U*^33^	*U*^12^	*U*^13^	*U*^23^
Br1A	0.0302 (5)	0.0275 (5)	0.0621 (8)	−0.0027 (4)	−0.0057 (5)	−0.0033 (5)
O1A	0.040 (4)	0.016 (3)	0.083 (6)	−0.009 (3)	0.007 (4)	−0.015 (3)
N1A	0.025 (4)	0.020 (4)	0.039 (5)	−0.005 (3)	−0.006 (3)	−0.001 (3)
N2A	0.022 (3)	0.020 (4)	0.043 (5)	−0.004 (3)	0.003 (3)	−0.008 (3)
C1A	0.029 (5)	0.023 (5)	0.043 (6)	−0.010 (4)	0.006 (4)	−0.003 (4)
C2A	0.023 (4)	0.021 (4)	0.033 (5)	−0.008 (3)	0.000 (4)	0.004 (4)
C3A	0.029 (5)	0.026 (5)	0.036 (5)	−0.010 (4)	−0.005 (4)	0.004 (4)
C4A	0.035 (5)	0.048 (6)	0.049 (6)	−0.022 (5)	−0.015 (5)	0.007 (5)
C5A	0.051 (6)	0.032 (5)	0.039 (6)	−0.030 (5)	−0.002 (5)	0.000 (4)
C6A	0.042 (5)	0.022 (4)	0.037 (6)	−0.013 (4)	−0.008 (4)	0.000 (4)
C7A	0.027 (4)	0.016 (4)	0.027 (5)	−0.008 (3)	−0.004 (4)	0.000 (3)
C8A	0.025 (4)	0.023 (4)	0.042 (6)	−0.004 (4)	0.004 (4)	−0.011 (4)
C9A	0.025 (4)	0.024 (4)	0.038 (5)	−0.012 (4)	0.001 (4)	−0.002 (4)
C10A	0.027 (4)	0.028 (5)	0.041 (6)	−0.011 (4)	−0.003 (4)	−0.002 (4)
C11A	0.033 (5)	0.059 (7)	0.038 (6)	−0.015 (5)	0.007 (5)	−0.013 (5)
C12A	0.066 (8)	0.098 (10)	0.021 (6)	−0.045 (8)	0.008 (5)	−0.007 (6)
C13A	0.062 (7)	0.061 (7)	0.049 (7)	−0.029 (6)	−0.016 (6)	0.018 (6)
C14A	0.041 (5)	0.032 (5)	0.038 (6)	−0.010 (4)	−0.009 (4)	0.010 (4)
C15A	0.033 (5)	0.028 (5)	0.027 (5)	−0.002 (4)	−0.005 (4)	−0.007 (4)
C16A	0.048 (6)	0.035 (5)	0.046 (7)	−0.012 (5)	−0.001 (5)	0.000 (5)
C17A	0.047 (6)	0.055 (7)	0.042 (7)	−0.015 (5)	0.002 (5)	−0.007 (5)
C18A	0.082 (10)	0.067 (9)	0.063 (9)	−0.023 (8)	0.022 (7)	−0.035 (7)
Br1B	0.0518 (7)	0.0338 (6)	0.0641 (8)	−0.0140 (5)	−0.0099 (6)	0.0050 (5)
O1B	0.037 (4)	0.019 (3)	0.064 (5)	−0.004 (3)	−0.009 (3)	−0.006 (3)
N2B	0.028 (4)	0.034 (5)	0.059 (6)	−0.004 (4)	−0.002 (4)	−0.011 (4)
N1B	0.038 (4)	0.032 (4)	0.026 (4)	0.000 (4)	−0.001 (3)	0.004 (3)
C1B	0.032 (5)	0.031 (5)	0.060 (7)	−0.012 (5)	−0.013 (5)	0.002 (5)
C2B	0.031 (5)	0.029 (5)	0.020 (5)	−0.004 (4)	−0.004 (4)	0.007 (4)
C3B	0.040 (5)	0.040 (6)	0.038 (6)	−0.013 (5)	−0.006 (4)	0.002 (4)
C4B	0.030 (5)	0.060 (7)	0.046 (7)	−0.016 (5)	−0.004 (5)	0.010 (5)
C5B	0.027 (5)	0.047 (6)	0.040 (6)	0.007 (4)	−0.004 (4)	0.007 (5)
C6B	0.038 (5)	0.029 (5)	0.045 (6)	−0.005 (4)	0.000 (5)	−0.007 (4)
C7B	0.029 (5)	0.024 (5)	0.038 (6)	−0.002 (4)	0.001 (4)	0.001 (4)
C8B	0.036 (5)	0.039 (6)	0.050 (7)	−0.014 (5)	−0.001 (5)	0.008 (5)
C9B	0.025 (5)	0.037 (5)	0.048 (6)	−0.005 (4)	−0.003 (4)	0.009 (5)
C10B	0.027 (5)	0.032 (5)	0.040 (6)	−0.002 (4)	0.001 (4)	0.001 (4)
C11B	0.045 (6)	0.046 (6)	0.051 (7)	−0.002 (5)	−0.001 (5)	−0.010 (5)
C12B	0.058 (8)	0.069 (9)	0.045 (7)	−0.006 (7)	−0.005 (6)	0.008 (6)
C13B	0.054 (7)	0.068 (8)	0.045 (7)	−0.010 (6)	−0.005 (6)	0.034 (6)
C14B	0.042 (6)	0.047 (7)	0.071 (9)	−0.015 (5)	−0.005 (6)	0.022 (6)
C15B	0.046 (6)	0.048 (6)	0.040 (6)	−0.014 (5)	−0.002 (5)	−0.007 (5)
C16B	0.070 (9)	0.110 (12)	0.056 (8)	−0.048 (9)	−0.012 (7)	−0.014 (8)
C17B	0.098 (11)	0.099 (11)	0.044 (8)	−0.048 (10)	−0.016 (8)	0.018 (7)
C18B	0.111 (13)	0.082 (11)	0.049 (9)	−0.020 (10)	0.018 (8)	−0.007 (7)

### Geometric parameters (Å, °)

**Table:**

Br1A—C10A	1.925 (9)	Br1B—C10B	1.910 (10)
O1A—C1A	1.224 (11)	O1B—C1B	1.212 (12)
N1A—C1A	1.333 (11)	N2B—C7B	1.354 (12)
N1A—C2A	1.440 (11)	N2B—C8B	1.427 (13)
N1A—C15A	1.452 (11)	N2B—H2BA	0.8800
N2A—C7A	1.353 (11)	N1B—C1B	1.305 (12)
N2A—C8A	1.452 (11)	N1B—C2B	1.443 (12)
N2A—H2AA	0.8800	N1B—C15B	1.509 (13)
C1A—H1AA	0.9500	C1B—H1B	0.9500
C2A—C3A	1.373 (12)	C2B—C3B	1.374 (13)
C2A—C7A	1.429 (12)	C2B—C7B	1.388 (13)
C3A—C4A	1.365 (13)	C3B—C4B	1.393 (15)
C3A—H3AA	0.9500	C3B—H3BA	0.9500
C4A—C5A	1.364 (14)	C4B—C5B	1.373 (16)
C4A—H4AA	0.9500	C4B—H4BA	0.9500
C5A—C6A	1.370 (13)	C5B—C6B	1.368 (14)
C5A—H5AA	0.9500	C5B—H5BA	0.9500
C6A—C7A	1.409 (12)	C6B—C7B	1.389 (13)
C6A—H6AA	0.9500	C6B—H6BA	0.9500
C8A—C9A	1.507 (13)	C8B—C9B	1.528 (15)
C8A—H8AA	0.9900	C8B—H8BA	0.9900
C8A—H8AB	0.9900	C8B—H8BB	0.9900
C9A—C14A	1.380 (12)	C9B—C10B	1.373 (14)
C9A—C10A	1.389 (12)	C9B—C14B	1.399 (14)
C10A—C11A	1.362 (14)	C10B—C11B	1.378 (15)
C11A—C12A	1.408 (17)	C11B—C12B	1.403 (17)
C11A—H11A	0.9500	C11B—H11B	0.9500
C12A—C13A	1.368 (17)	C12B—C13B	1.375 (19)
C12A—H12A	0.9500	C12B—H12B	0.9500
C13A—C14A	1.391 (15)	C13B—C14B	1.396 (17)
C13A—H13A	0.9500	C13B—H13B	0.9500
C14A—H14A	0.9500	C14B—H14B	0.9500
C15A—C16A	1.505 (14)	C15B—C16B	1.502 (16)
C15A—H15A	0.9900	C15B—H15C	0.9900
C15A—H15B	0.9900	C15B—H15D	0.9900
C16A—C17A	1.520 (14)	C16B—C17B	1.550 (18)
C16A—H16A	0.9900	C16B—H16C	0.9900
C16A—H16B	0.9900	C16B—H16D	0.9900
C17A—C18A	1.478 (17)	C17B—C18B	1.47 (2)
C17A—H17A	0.9900	C17B—H17C	0.9900
C17A—H17B	0.9900	C17B—H17D	0.9900
C18A—H18A	0.9800	C18B—H18D	0.9800
C18A—H18B	0.9800	C18B—H18E	0.9800
C18A—H18C	0.9800	C18B—H18F	0.9800
			
C1A—N1A—C2A	119.2 (8)	C7B—N2B—C8B	124.9 (8)
C1A—N1A—C15A	121.5 (8)	C7B—N2B—H2BA	117.6
C2A—N1A—C15A	119.2 (7)	C8B—N2B—H2BA	117.6
C7A—N2A—C8A	123.7 (7)	C1B—N1B—C2B	122.6 (8)
C7A—N2A—H2AA	118.1	C1B—N1B—C15B	118.8 (9)
C8A—N2A—H2AA	118.1	C2B—N1B—C15B	118.6 (7)
O1A—C1A—N1A	127.6 (10)	O1B—C1B—N1B	128.1 (11)
O1A—C1A—H1AA	116.2	O1B—C1B—H1B	115.9
N1A—C1A—H1AA	116.2	N1B—C1B—H1B	115.9
C3A—C2A—C7A	120.1 (8)	C3B—C2B—C7B	122.3 (9)
C3A—C2A—N1A	121.0 (8)	C3B—C2B—N1B	118.4 (9)
C7A—C2A—N1A	118.9 (7)	C7B—C2B—N1B	119.3 (8)
C4A—C3A—C2A	122.2 (9)	C2B—C3B—C4B	120.2 (10)
C4A—C3A—H3AA	118.9	C2B—C3B—H3BA	119.9
C2A—C3A—H3AA	118.9	C4B—C3B—H3BA	119.9
C5A—C4A—C3A	118.5 (9)	C5B—C4B—C3B	118.2 (9)
C5A—C4A—H4AA	120.8	C5B—C4B—H4BA	120.9
C3A—C4A—H4AA	120.8	C3B—C4B—H4BA	120.9
C4A—C5A—C6A	122.0 (9)	C6B—C5B—C4B	121.0 (10)
C4A—C5A—H5AA	119.0	C6B—C5B—H5BA	119.5
C6A—C5A—H5AA	119.0	C4B—C5B—H5BA	119.5
C5A—C6A—C7A	121.0 (9)	C5B—C6B—C7B	122.2 (10)
C5A—C6A—H6AA	119.5	C5B—C6B—H6BA	118.9
C7A—C6A—H6AA	119.5	C7B—C6B—H6BA	118.9
N2A—C7A—C6A	124.0 (8)	N2B—C7B—C2B	121.0 (8)
N2A—C7A—C2A	119.8 (7)	N2B—C7B—C6B	122.9 (9)
C6A—C7A—C2A	116.2 (8)	C2B—C7B—C6B	116.2 (9)
N2A—C8A—C9A	115.2 (8)	N2B—C8B—C9B	116.8 (8)
N2A—C8A—H8AA	108.5	N2B—C8B—H8BA	108.1
C9A—C8A—H8AA	108.5	C9B—C8B—H8BA	108.1
N2A—C8A—H8AB	108.5	N2B—C8B—H8BB	108.1
C9A—C8A—H8AB	108.5	C9B—C8B—H8BB	108.1
H8AA—C8A—H8AB	107.5	H8BA—C8B—H8BB	107.3
C14A—C9A—C10A	116.1 (9)	C10B—C9B—C14B	116.6 (10)
C14A—C9A—C8A	121.9 (8)	C10B—C9B—C8B	122.2 (9)
C10A—C9A—C8A	122.0 (8)	C14B—C9B—C8B	121.2 (10)
C11A—C10A—C9A	123.6 (9)	C9B—C10B—C11B	123.8 (10)
C11A—C10A—Br1A	118.2 (7)	C9B—C10B—Br1B	119.0 (8)
C9A—C10A—Br1A	118.1 (7)	C11B—C10B—Br1B	117.2 (8)
C10A—C11A—C12A	118.6 (10)	C10B—C11B—C12B	119.6 (12)
C10A—C11A—H11A	120.7	C10B—C11B—H11B	120.2
C12A—C11A—H11A	120.7	C12B—C11B—H11B	120.2
C13A—C12A—C11A	119.8 (10)	C13B—C12B—C11B	117.3 (12)
C13A—C12A—H12A	120.1	C13B—C12B—H12B	121.3
C11A—C12A—H12A	120.1	C11B—C12B—H12B	121.3
C12A—C13A—C14A	119.5 (11)	C12B—C13B—C14B	122.4 (11)
C12A—C13A—H13A	120.2	C12B—C13B—H13B	118.8
C14A—C13A—H13A	120.2	C14B—C13B—H13B	118.8
C9A—C14A—C13A	122.4 (10)	C13B—C14B—C9B	120.2 (12)
C9A—C14A—H14A	118.8	C13B—C14B—H14B	119.9
C13A—C14A—H14A	118.8	C9B—C14B—H14B	119.9
N1A—C15A—C16A	113.6 (8)	C16B—C15B—N1B	109.8 (9)
N1A—C15A—H15A	108.9	C16B—C15B—H15C	109.7
C16A—C15A—H15A	108.9	N1B—C15B—H15C	109.7
N1A—C15A—H15B	108.9	C16B—C15B—H15D	109.7
C16A—C15A—H15B	108.9	N1B—C15B—H15D	109.7
H15A—C15A—H15B	107.7	H15C—C15B—H15D	108.2
C15A—C16A—C17A	116.0 (8)	C15B—C16B—C17B	109.6 (11)
C15A—C16A—H16A	108.3	C15B—C16B—H16C	109.8
C17A—C16A—H16A	108.3	C17B—C16B—H16C	109.8
C15A—C16A—H16B	108.3	C15B—C16B—H16D	109.8
C17A—C16A—H16B	108.3	C17B—C16B—H16D	109.8
H16A—C16A—H16B	107.4	H16C—C16B—H16D	108.2
C18A—C17A—C16A	114.6 (10)	C18B—C17B—C16B	113.4 (12)
C18A—C17A—H17A	108.6	C18B—C17B—H17C	108.9
C16A—C17A—H17A	108.6	C16B—C17B—H17C	108.9
C18A—C17A—H17B	108.6	C18B—C17B—H17D	108.9
C16A—C17A—H17B	108.6	C16B—C17B—H17D	108.9
H17A—C17A—H17B	107.6	H17C—C17B—H17D	107.7
C17A—C18A—H18A	109.5	C17B—C18B—H18D	109.5
C17A—C18A—H18B	109.5	C17B—C18B—H18E	109.5
H18A—C18A—H18B	109.5	H18D—C18B—H18E	109.5
C17A—C18A—H18C	109.5	C17B—C18B—H18F	109.5
H18A—C18A—H18C	109.5	H18D—C18B—H18F	109.5
H18B—C18A—H18C	109.5	H18E—C18B—H18F	109.5
			
C2A—N1A—C1A—O1A	−176.4 (8)	C2B—N1B—C1B—O1B	−174.7 (9)
C15A—N1A—C1A—O1A	−0.5 (14)	C15B—N1B—C1B—O1B	7.6 (15)
C1A—N1A—C2A—C3A	67.9 (11)	C1B—N1B—C2B—C3B	72.6 (12)
C15A—N1A—C2A—C3A	−108.1 (9)	C15B—N1B—C2B—C3B	−109.6 (10)
C1A—N1A—C2A—C7A	−113.6 (9)	C1B—N1B—C2B—C7B	−107.1 (11)
C15A—N1A—C2A—C7A	70.4 (10)	C15B—N1B—C2B—C7B	70.7 (11)
C7A—C2A—C3A—C4A	−0.1 (14)	C7B—C2B—C3B—C4B	−1.2 (15)
N1A—C2A—C3A—C4A	178.3 (8)	N1B—C2B—C3B—C4B	179.1 (9)
C2A—C3A—C4A—C5A	−0.8 (14)	C2B—C3B—C4B—C5B	0.1 (15)
C3A—C4A—C5A—C6A	0.4 (15)	C3B—C4B—C5B—C6B	0.9 (16)
C4A—C5A—C6A—C7A	0.8 (14)	C4B—C5B—C6B—C7B	−0.8 (16)
C8A—N2A—C7A—C6A	6.4 (14)	C8B—N2B—C7B—C2B	−173.3 (9)
C8A—N2A—C7A—C2A	−173.7 (8)	C8B—N2B—C7B—C6B	6.0 (16)
C5A—C6A—C7A—N2A	178.2 (9)	C3B—C2B—C7B—N2B	−179.3 (9)
C5A—C6A—C7A—C2A	−1.6 (12)	N1B—C2B—C7B—N2B	0.4 (13)
C3A—C2A—C7A—N2A	−178.6 (8)	C3B—C2B—C7B—C6B	1.4 (14)
N1A—C2A—C7A—N2A	2.9 (12)	N1B—C2B—C7B—C6B	−179.0 (8)
C3A—C2A—C7A—C6A	1.3 (12)	C5B—C6B—C7B—N2B	−179.7 (10)
N1A—C2A—C7A—C6A	−177.2 (8)	C5B—C6B—C7B—C2B	−0.4 (15)
C7A—N2A—C8A—C9A	−80.4 (10)	C7B—N2B—C8B—C9B	−80.8 (12)
N2A—C8A—C9A—C14A	−2.5 (12)	N2B—C8B—C9B—C10B	177.2 (9)
N2A—C8A—C9A—C10A	178.9 (8)	N2B—C8B—C9B—C14B	−3.6 (14)
C14A—C9A—C10A—C11A	−2.0 (13)	C14B—C9B—C10B—C11B	−2.4 (14)
C8A—C9A—C10A—C11A	176.7 (9)	C8B—C9B—C10B—C11B	176.9 (9)
C14A—C9A—C10A—Br1A	−179.1 (6)	C14B—C9B—C10B—Br1B	−180.0 (7)
C8A—C9A—C10A—Br1A	−0.3 (11)	C8B—C9B—C10B—Br1B	−0.7 (12)
C9A—C10A—C11A—C12A	0.3 (15)	C9B—C10B—C11B—C12B	1.6 (16)
Br1A—C10A—C11A—C12A	177.4 (8)	Br1B—C10B—C11B—C12B	179.3 (8)
C10A—C11A—C12A—C13A	1.1 (16)	C10B—C11B—C12B—C13B	−1.2 (17)
C11A—C12A—C13A—C14A	−0.8 (17)	C11B—C12B—C13B—C14B	1.8 (18)
C10A—C9A—C14A—C13A	2.4 (14)	C12B—C13B—C14B—C9B	−2.6 (18)
C8A—C9A—C14A—C13A	−176.4 (9)	C10B—C9B—C14B—C13B	2.8 (15)
C12A—C13A—C14A—C9A	−1.1 (17)	C8B—C9B—C14B—C13B	−176.5 (10)
C1A—N1A—C15A—C16A	−106.0 (9)	C1B—N1B—C15B—C16B	−93.1 (12)
C2A—N1A—C15A—C16A	69.9 (10)	C2B—N1B—C15B—C16B	89.0 (12)
N1A—C15A—C16A—C17A	69.3 (11)	N1B—C15B—C16B—C17B	176.4 (11)
C15A—C16A—C17A—C18A	66.8 (14)	C15B—C16B—C17B—C18B	−61.8 (18)

### Hydrogen-bond geometry (Å, °)

**Table:**

*D*—H···*A*	*D*—H	H···*A*	*D*···*A*	*D*—H···*A*
N2A—H2AA···O1A^i^	0.88	2.15	2.947 (9)	150
N2B—H2BA···O1B^ii^	0.88	2.18	3.020 (10)	159

Symmetry codes: (i) −*x*+2, −*y*+1, −*z*+1; (ii) −*x*, −*y*+2, −*z*+2.
